# Gender differences in work attendance among health care workers in Northern Nigeria during the COVID-19 pandemic

**DOI:** 10.1016/j.eclinm.2022.101605

**Published:** 2022-08-03

**Authors:** Modupe Taiwo, Oluwatoyin Oyekenu, Ferdinard Ekeh, Arnab K. Dey, Anita Raj

**Affiliations:** a Save the Children, Sierra Leone; b Global Alliance for Improved Nutrition, Nigeria; c Palladium, Nigeria; d Center on Gender Equity and Health, Department of Medicine, University of California, San Diego, USA; e Department of Education Studies, Division of Social Sciences, University of California, San Diego, USA

**Keywords:** Health workers, COVID -19, Northern nigeria, Gender, Household burden

## Abstract

**Background:**

The COVID-19 pandemic has resulted in the overwork of health care workers (HCWs) and greater household burdens for women. This study examines gender differences in HCWs' work attendance under COVID-19 and household burdens as a potential mediator of the gender difference in Northern Nigeria.

**Methods:**

From April to May 2021, we conducted a cross-sectional survey on work and household burdens with a convenience sample of male and female HCWs (*N*=334) across 16 facilities in the Gombe, Katsina, and Zamfara states in Northern Nigeria. We used a series of multilevel modified Poisson regression models to examine the associations between gender and HCW work attendance. We also tested the mediation effect of household burdens on this observed association.

**Findings:**

Only 2·10% of HCWs reported <5 days of work in a typical week; 35·33% worked 6-7 days a week (i.e., HCW overwork). Males were more likely than females to report HCW overwork (46·33% vs. 22·93%), and females were more likely than males to report an increase in household burden (59·24% vs. 40·68%). Adjusted regression models found that men were more likely than women to report HCW overwork (ARR: 1·76, 95% CI: 1·17-2·66). Increased household burdens mediated 9 percent of the total effect between gender and HCW work attendance.

**Interpretation:**

The COVID-19 pandemic in Northern Nigeria made female HCWs contend with the dual burdens of formal and informal care work. This contributes to lower attendance among female HCWs and overwork for their male counterparts.

**Funding:**

Bill and Melinda Gates Foundation Grant Numbers: OPP1163682 & INV018007.

**Research in context**

**Evidence before this study**

In March 2022, we conducted a PubMed review of the literature on gender differences in healthcare worker (HCW) work attendance under the COVID-19 pandemic. We used the search terms “pandemic” or “COVID” and “health worker” or “healthcare worker” and “gender” or “sex” and limited our search to systematic reviews ever published on these topics. This search generated 79 papers, almost all of which focused on gender differences in health outcomes of HCWs and demonstrated adverse mental health effects, partially due to household burdens and gendered stress for females compared with males. This work also showed higher infection rates due to greater engagement in frontline care but lower fatality rates for females than male HCWs. This literature offered limited data from low- and middle-income (LMIC) settings and Africa. We found no papers on gender differences in HCW work attendance during COVID crises in LMICs, though the papers on health outcomes demonstrated likely impacts on work attendance.

**Added value of this study**

This study adds to the value of our understanding of the gendered effects of COVID-19 on HCWs in Africa by examining gender differences in work attendance and increased household burdens as a potential mediator of observed gender differences among full-time HCWs in Northern Nigeria who served during the COVID-19 pandemic. Findings from our study show high work attendance than the standard work week of five days for males and females. However, male HCWs are more likely than females to report overwork (6-7 days of work per week). Greater household burdens that female HCWs’ experienced relative to men during the pandemic partially explain the observed gender difference in work attendance.

**Implications of all the available evidence**

In Northern Nigeria, the COVID-19 pandemic resulted in the overwork of male HCWs. Increased household burdens that disproportionately affected female HCWs partially explain the unequal gender distribution of overwork. Concrete domestic labor support from health systems, such as meals and childcare, may be necessary for ensuring access to the female HCWs’ labor. There is also an urgent need for shifts in social norms to create more equitable household labor solutions and value for women's work to ensure women's effective participation in providing healthcare services. At the same time, there is a need to bolster health systems to reduce reliance on overwork from HCWs regardless of their gender.

Alt-text: Unlabelled box

## Introduction

The Coronavirus-2019 (COVID-19) has profoundly impacted the social life, health care system, economy, and well-being across the globe, and particularly in Nigeria. By the end of February 2022, Nigeria reported over 254,000 cases and 3142 deaths.^1^ Since the onset of COVID-19, several policies and interventions have been proposed, implemented, and enforced to prevent, mitigate and control the direct and indirect impacts of the virus on the population. In a rapidly changing health care environment and case definition of the different variants of the virus, healthcare workers (HCW) are at the forefront in the fight against the pandemic. In this context, HCWs are experiencing infection, burnout, and even mistreatment,^2^ with female health workers being disproportionately impacted.^3^, ^4^, ^5^, ^6^ However, much of the understanding of these issues for female HCWs comes from high-income countries and Asia, with relatively scant literature emerging from low- and middle-income country (LMIC) settings and Africa.

Before the pandemic, Nigeria struggled with shortages in the healthcare workforce and a lack of infrastructure and equipment to support optimum care. In addition, huge disparities existed in the availability of the healthcare workforce depending on the level and location of the health facilities.^7^ The pandemic only added to the existing strains on the country's health system.^8^ The shortages in supplies and the inequitable distribution of healthcare workers in Nigeria have been shown to result in work-related stress, affecting HCW's work-life balance, job satisfaction, and productivity.^9^^,^^10^ In such a context, psychosocial care, and infection management for HCWs are far from adequate, compromising the safety of the workforce.

According to the World Health Organization, 70% of the global health workforce is female, which some describe as a feminization of healthcare jobs.^11^ In Nigeria, women constitute the majority of medical doctors and nursing and midwifery personnel, with data from 2018 reporting that 65% of medical doctors and 87% of nursing personnel were women.^11^ However, in Northern Nigeria, trained HCWs on the frontlines are majorly male due to low levels of education among girls, though untrained HCWs are more likely to be female.^12^ Correspondingly, much of the personal protective equipment designed to reduce COVID-19 exposure for HCWs were more suitable for men by design,^13^ which may increase female HCWs’ risk of infection and discourage them to extend work hours.^14^ Additionally, research has also shown greater mental health impacts of the pandemic on female HCWs compared to their male counterparts.^15^ Again, much of our understanding of these issues comes from high-income settings, with very little data available on the work situation of HCWs in Nigeria during the COVID-19 pandemic.

In addition to the growing burdens and risks faced at work during the pandemic, female HCWs also face increased domestic responsibilities, including childcare, children's education due to school closure, and higher cooking and cleaning demands with family members home full-time during shutdowns.^16^ The demands to meet these gender role expectations may help explain why female HCWs are experiencing higher stress and poorer physical and psychological health outcomes than their male counterparts. In a study with health workers on gender and race, Wenham et al. concluded that female HCWs, particularly those of minority racial/ethnic heritage, face the downstream effects of care work manifesting in distress, burnout, and psychosomatic stress disorders.^17^ Correspondingly, we see a significant deterioration of HCWs' quality of life and the ability to provide optimum health service delivery across several countries.^18^ In such contexts, many women drop out of the workforce, though research has not examined this specific concern related to gender differences in job loss for health workers.^19^^,^^20^ Nonetheless, we have seen a loss of HCWs due to infection and death on a global scale,^6^^,^^21^ and due to abuse and burnout, at least in some countries, such as the United States.^22^

A review of the literature on gender and COVID-19 in Nigeria shows similar patterns that are seen globally, with women more likely than men to experience job loss,^23^ increase in household burdens, and mental health distress.^24^^,^^25^ As mentioned above, this research was not specific to HCWs; however, research conducted before the pandemic indicates that gendered effects on work are notable among HCWs. A pre-pandemic study conducted in Southern Nigeria found that female HCWs were more likely to report work-related stress and burnout than their male counterparts.^26^ Similarly, pre-pandemic studies from Northern Nigeria, characterized by more traditional gender role norms than those seen in the South, found that female HCWs were more than three times as likely as male HCWs to have work-related depression.^27^ Such findings suggest that gendered effects of COVID-19 are likely a concern for HCWs in Nigeria, hindering women's work as HCWs. These concerns may be even more severe in Northern Nigeria, where there are protracted conflicts, internal displacement, impeded education for girls, and high rates of gender-based violence.^28^

In this paper, we examined gender differences in household burden and work attendance among HCWs in three states in Northern Nigeria, a region already underserved by the health care system and contending with ongoing instability and insecurity. We then assessed whether household burden mediates gender effects on work attendance among these HCWs. These findings highlight whether addressing issues of household burden for women HCWs can potentially support their retention at work.

## Methods

### Data collection

From April to May 2021, we collected cross-sectional survey data from a convenience sample of full-time employed HCWs (*N*=334) at primary, secondary, and tertiary health facilities in Gombe, Katsina, and Zamfara states in Northern Nigeria. We included all available providers at health facilities providing COVID-19 care in these states (*N*=16 facilities).

Our survey team comprised fifteen interviewers under three supervisors. The survey team received three days of training focused on the study's aims, research protocols, and data collection instruments and discussed the logistics of data collection. The training also included guidance on COVID-19 infection prevention and protocols to reduce the risk for infection and transmission during research activities. These included hand sanitization before and after each interview, wearing masks during the survey, and maintaining social distancing during data collection. After the initial three-day training, the teams piloted the survey instrument in nearby health facilities outside those designated for the actual study. After the pilot, the teams converged for a debriefing session where they shared their feedback on the survey instrument and the data collection process, based on which we revised the study instrument. This process did not lead to any major structural change to the study tool except that we expanded the list of the healthcare workforce to include positions that were missing in the earlier versions. The survey teams also requested extra face masks and hand sanitizers for the respondents, as many health facilities experienced stock out of these supplies. The State Research Ethics Committees and the Institutional Research Review boards in all the Federal medical health institutions in each state provided ethical approval for the study. (Zamfara: ZSHREC01032021, Katsina: MOH/Adm/SUB/1152/1/427 and Gombe: MOH/ADM/621/V.1/299).

Written consents were obtained from all respondents after the study aims had been explained in both English and Hausa language to ensure understanding of the participation in the study.

### Measures

#### Dependent variable

We used healthcare workers' self-reported attendance during the pandemic as the dependent variable. We initially intended to have a categorical variable indicating whether respondents worked less than the standard workweek (<5 days), the standard workweek (5 days), or HCW overwork (6-7 days). However, only 2·10% of the sample reported working less than the standard workweek. Hence, we coded respondents who reported that they attended to their duties in the health facility for more than five days per week during the pandemic as ‘1’, defining this as HCW overwork, and those who reported working for five days or less in a week as ‘0’.

#### Independent variable

We used the gender of HCWs (male/female) as the primary independent variable.

#### Mediator

We captured HCWs' burden of household work using the following items: “My tasks at home, such as house cleaning, cooking, taking care of children, and washing clothes and plates, have increased” and “I do most of the house-work and do not get assistance from anyone during the COVID-19 pandemic.” We adapted these items from one of our previous studies conducted before the COVID-19 pandemic. The study included items on the household burden for Community Health Workers in India and studied the effect of community and family support on their productivity.^29^ Both the items had 5-point Likert-type response options ranging from “Strongly Agree” to “Strongly Disagree.” We coded those reporting “Strongly agree” or “Agree” to both the items as ‘1’, indicating an increased burden of household work during the pandemic that respondents bore; we coded all other cases as ‘0’.

#### Covariates

We considered covariates related to demographics (age- categorized as 21-30, 31-40, 41-50, >50 years; currently married- yes/no, work experience (categorized as <6, 6-9, 10+ years), and provider cadre (categorized as nurse/midwife, doctor, community health worker [CHW] or other). We also included the type of facility (primary, secondary, or tertiary) and the state where the facilities were located.

### Data analysis

We performed descriptive analysis to assess the independent variable, mediator, covariates, and dependent variable frequencies. We cross-tabulated all variables by our dependent variable, work attendance (which we will define for the remainder of the paper as HCW overwork). We also cross-tabulated all variables by our independent variable, gender.

We used a series of multilevel modified Poisson regression models with random intercepts at the health facility level. We used modified Poisson regressions instead of logistic regression models since our outcome is non-rare. In cases of non-rare binary outcomes, the odds ratios derived from logistic regression models strongly overestimate risk ratios,^30^ and the use of modified Poisson regression (i.e. Poisson regression with robust error variance) to estimate risk ratios is recommended.^31^

We first developed a simple regression model to assess the total effect of gender on HCW overwork (Model-1). We then conducted the same model and additionally adjusted for the increased burden of household work during the pandemic (Model-2). We then developed a model to examine the association between HCWs' gender and the increased burden of household work during the pandemic (Model-3).

Given the number of covariates we wanted to consider and the relatively small sample size, we used the backward selection method for variable selection.^32^^,^^33^ We use the step() function available in the stats package in R for this. The algorithm starts with all the covariates and drops them one step at a time to identify the combination of covariates that yields the minimum Akaike Information Criteria (AIC). This approach identified *provider cadre* and the *state* where the health facilities were located as the covariates to be retained in the model, in addition to the independent variable and the mediator. We adjusted for these variables in all the models described above.

Finally, we performed causal mediation analyses to quantify the mediating effect of the increased burden of household work during the pandemic on the association between gender and HCW overwork. We used the regression-based approach under the potential outcomes framework to study mediation.^34^^,^^35^ We first estimated the Natural Direct Effect (NDE) and Natural Indirect Effect (NIE) for binary mediator and outcome and computed the 95% Confidence Interval around NIE using bootstrapping with 5000 replications.^35^^,^^36^ Finally, we estimated the proportion of total effect between gender and attendance mediated by the burden of household work.^37^

### Role of the funding source

The donor providing funding for this work has no involvement with the study design; collection, analysis, and interpretation of data; writing of the report, or decision to submit the paper for publication.

## Results

### Sample characteristics

Almost half of the participants were female (47·01%). Half of the participants reported that they were a nurse/midwife (50·60%), 12·57% were doctors, and 12·87% were community health workers (Table 1). Female HCWs were more likely than male HCWs to be a nurse/midwives (62·42% vs. 40·11%), whereas male HCWs were more likely than female HCWs to be a doctor (20·34% vs. 3·82%) (Table 2). HCW overwork (6-7 day workweek) was reported by a third of HCWs (35·33%), with males more likely than females to report HCW overwork (46·33% vs. 22·93%). Half of the respondents (49·40%) reported an increase in household burden during the pandemic, with females more likely than their male counterparts to report this (59·24% vs. 40·68%).

**Table 1:** Descriptive statistics of healthcare workers’ attendance, burden of household work, gender, and other covariates (*N* = 334).

		Overall sample (*N* = 334)	Attendance in health facilities during the COVID-19 pandemic	
			Five days or less (*N* = 216)	More than 5 days (*N* = 118)
		%	%	%
Work Attendance in health facilities during the COVID-19 pandemic (HCW overwork)	Five days or less	64·67		
	More than five days	35·33		
The burden of household work increased during the pandemic	No	50·60	44·44	61·86
	Yes	49·40	55·56	38·14
Gender	Female	47·01	56·02	30·51
	Male	52·99	43·98	69·49
Age	21 to 30 years	17·37	15·28	21·19
	31 to 40 years	52·40	53·70	50·00
	41 to 50 years	23·05	23·61	22·03
	51 years or more	7·19	7·41	6·78
Years of experience	Five years or less	24·85	21·76	30·51
	6 to 9 years	32·93	33·33	32·20
	Ten years or more	42·22	44·91	37·29
Marital status	Married	81·14	80·56	82·20
	Single/Divorced or Separated	18·86	19·44	17·80
Provider Cadre	Nurse / Midwife	50·60	58·80	35·59
	Doctor	12·57	9·26	18·64
	Community Health Worker	12·87	13·89	11·02
	Other health service provider	23·95	18·06	34·75
Facility type	Primary	10·78	10·65	11·02
	Secondary	23·35	20·83	27·97
	Tertiary	65·87	68·52	61·02
State	Gombe	33·83	31·48	38·14
	Katsina	30·24	26·39	37·29
	Zamfara	35·93	42·13	24·58

**Table 2:** Descriptive statistics across gender of healthcare workers (*N* = 334).

		Overall sample (*N* = 334)	Gender of healthcare worker	
			Female (*N* = 157)	Male (*N* = 177)
		%	%	%
Work Attendance in health facilities during the COVID-19 pandemic (HCW overwork)	Five days or less	64·67	77·07	53·67
	More than five days	35·33	22·93	46·33
The burden of household work increased during the pandemic	No	50·60	40·76	59·32
	Yes	49·40	59·24	40·68
Age	21 to 30 years	17·37	21·02	14·12
	31 to 40 years	52·40	51·59	53·11
	41 to 50 years	23·05	19·75	25·99
	51 years or more	7·19	7·64	6·78
Years of experience	Five years or less	24·85	26·11	23·73
	6 to 9 years	32·93	28·03	37·29
	Ten years or more	42·22	45·86	38·98
Marital status	Married	81·14	79·62	82·49
	Single/Divorced or Separated	18·86	20·38	17·51
Provider cadre	Nurse / Midwife	50·60	62·42	40·11
	Doctor	12·57	3·82	20·34
	Community Health Worker	12·87	14·01	11·86
	Other health service provider	23·95	19·75	27·68
Facility type	Primary	10·78	10·83	10·73
	Secondary	23·35	24·84	22·03
	Tertiary	65·87	64·33	67·23
State	Gombe	33·83	31·85	35·59
	Katsina	30·24	32·48	28·25
	Zamfara	35·93	35·67	36·16

### Multilevel models exploring the associations between HCW gender, increased household burden, and HCW overwork during the pandemic

Our multilevel models fitted with random intercepts at the health facility level showed that the facility level random effects were very close to zero. This indicates that individual providers accounted for most of the variance in the outcome. Male HCWs were significantly more likely to report HCW overwork in our adjusted model (Model 1), which accounted for demographics and provider cadre (ARR: 1·76, 95% CI: 1·17, 2·66). Doctors were also more likely than nurses/midwives to report HCW overwork in this adjusted model (ARR: 1·82, 95% CI: 1·05, 3·16) (Table 3). In our model, additionally adjusting for increased household burden (Model 2), the effect of gender was maintained, but the increased household burden was not significantly associated with our outcome (ARR: 0·71, 95% CI: 0·48, 1·05) (Table 4). Our last model that explored the association between HCW gender and increased household burden (Model 3) shows that male HCWs were significantly less likely than their female counterparts to report increased household burden during the COVID-19 pandemic (ARR: 0·68, 95% CI: 0·49, 0·93) (Table 5).

**Table 3:** Model 1: Association between gender and healthcare workers’ attendance (HCW overwork) during the pandemic (*N* = 334).

		Adjusted risk ratio	Robust 95% LCI	Robust 95% UCI
Gender	Female	Ref	-	-
	Male	1·76	1·17	2·66
Provider cadre	Nurse / Midwife	Ref	-	-
	Doctor	1·82	1·05	3·16
	Community Health Worker	1·03	0·53	1.97
	Other health service provider	1·60	1·02	2·50
State	Gombe	Ref	-	-
	Katsina	1·01	0·64	1·57
	Zamfara	0·59	0·35	0·99

Model fitted with random intercept at the health facility level.

**Table 4:** Model 2: Association between healthcare workers’ increased burden of household work and their work attendance (HCW overwork) during the pandemic (*N* = 334).

		Adjusted risk ratio	Robust 95% LCI	Robust 95% UCI
The burden of household work increased during the pandemic	No	Ref	-	-
	Yes	0·71	0·48	1·05
Gender	Female	Ref	-	-
	Male	1·66	1·10	2·52
Provider cadre	Nurse / Midwife	Ref	-	-
	Doctor	1·84	1·06	3·19
	Community Health Worker	1·02	0·53	1·97
	Other health service provider	1·56	0·99	2·45
State	Gombe	Ref	-	-
	Katsina	0·90	0·57	1·44
	Zamfara	0·56	0·33	0·94

Model fitted with random intercept at the health facility level.

**Table 5:** Model 3: Association between gender and healthcare workers’ increased burden of household work during the pandemic (*N* = 334).

		Adjusted risk ratio	Robust 95% LCI	Robust 95% UCI
Gender	Female	Ref	-	-
	Male	0·68	0·49	0·93
Provider cadre	Nurse / Midwife	Ref	-	-
	Doctor	1·01	0·58	1·76
	Community Health Worker	1·00	0·63	1·59
	Other health service provider	0·90	0·60	1·37
State	Gombe	Ref	-	-
	Katsina	0·53	0·34	0·81
	Zamfara	0·77	0·53	1·13

Model fitted with random intercept at the health facility level.

### Mediation analysis

Our mediation analysis shows that adding an increased burden of household work to Model 1 reduced the effect of Gender on HCW overwork, suggesting a partial mediating effect. Indeed, we found a significant indirect effect of an increased household burden on the association between gender and HCW overwork (ARR = 1.02, 95% CI: 1.01, 1.04). We also found that increased household work explains 8.57% of the total effect of gender on HCW overwork.

## Discussion

There has been a paucity of research on the impact of the COVID-19 pandemic on healthcare workers (HCWs) in places such as Northern Nigeria, a low-resource region with an already strained health system prior to the pandemic. Global research shows that HCWs labor and health risks have only escalated under the pandemic,^2^ and female HCWs have additionally faced a greater increase in household labor responsibilities, as the number of family members at home increased and school closures created parental education responsibilities.^16^ This study offers an understanding of these issues among HCWs in Northern Nigeria by examining gender differences between work attendance and household work burdens in this population. Importantly, we found that 98% of HCWs reporting working at least their full work week of 5 days, demonstrating the commitment of these HCWs to provide care regardless of gender. However, we did find that more than one-third of HCWs reported an HCW overwork in a typical week, extending their workweek by one to two days under the pandemic, with males more likely to do this than females. Overwork should not be the goal for fatigued HCWs, but these findings suggest that it was a demand during the pandemic for the health workers.

While women were less likely than men to report HCW overwork, they were more likely to have experienced increased household burdens under the pandemic, and these household burdens explain 9 percent of the effect of gender on HCW overwork. In other words, both male and female HCWs are contending with overwork, males by extended time in the HCW space, whereas females by the double burden of home and work responsibilities. For male and female HCWs, quality of life and quality of care are likely affected by this overwork, as seen across other national contexts.^18^ Importantly, unequal access to HCWs by gender also impedes the nature of care available. Nurses are the largest provider cadre and are more likely to be female. Recent research from Nigeria found that stress and turnover are a concern. Support for work-life balance, improved pay, and increasing the HCW labor force is recommended to strengthen the system.^38^^,^^39^

These findings correspond to a global study conducted prior to the pandemic, which also showed that male HCWs work more hours and further demonstrated that this was associated with their higher compensation, leadership positioning, and control over work hours.^19^^,^^40^ Findings also highlight that women's unequal household burdens contribute to these ongoing inequities, which not only compromise women HCWs' labor force participation and income generation but also strain health care systems in times of crisis. Such strain can be a particular concern in lower development contexts such as Northern Nigeria, where health systems were already unable to meet the population's needs.^12^ At the same time, findings also demonstrate that the strained health system under COVID called for extended workweeks from providers. We need to bolster healthcare systems with an increase in the number of healthcare workers so that they are better equipped to manage crises.

Over the past twenty years, women's representation and participation in health care have increased significantly, particularly in the frontlines of care,^11^^,^^40^ including in contexts such as Northern Nigeria, where women and girls have had lesser educational and occupational opportunities.^12^ However, women's domestic roles and responsibilities remain largely unchanged, with women continuing to bear the greater burden of household labor.^41^ The COVID-19 pandemic has added to both household burdens and work stress. Women, particularly women in the care economy, disproportionately experience these burdens and stresses.^42^^,^^43^ While prior work with HCWs has emphasized greater mental health burdens for females compared with male HCWs under the pandemic; these findings extend that work and highlight domestic burdens as potentially affecting women HCWs' labor force participation.^44^ Lesser hours from female HCWs as a consequence of domestic labor responsibilities can be a particular cost for health care in Africa, where HCW migration is actively occurring to fill HCW shortages around the globe.^45^ Ensuring strength and stability of health care systems in Africa, especially in low resource areas such as Northern Nigeria, will need to improve the support available to female HCWs to facilitate their longer term and full time engagement as a care provider. To this end, domestic labor support may be valuable to facilitate women HCWs retention and hours in service, in addition to shift in social norms to promote more equitable domestic labor distribution.

While this study offers important insights into the gendered risks to the health care workforce under COVID-19 and the opportunities to address these risks through more gender transformative approaches such as social norm change related to household labor, findings should be considered in light of certain study limitations. This is a cross-sectional study in a region of Northern Nigeria, such that causality cannot be presumed from these findings, and generalizability is limited to health providers in this low-resource area. We also rely on self-report data from survey data, which may be subject to social desirability and recall bias. Our sample size is not large, with small cell sizes by provider cadre, limiting our understanding within cadre groups. Nonetheless, we did adjust by cadre to gain insight into what cadres are more likely to overwork.

This study was a one-time survey conducted in response to COVID-19 when health facilities and healthcare providers struggled to provide care to a deluge of COVID-19 patients. Our primary purpose of data collection was to gather programmatic insights that could inform our strategies to engage with healthcare providers during the crisis. As such, we kept our survey questions brief as we did not want to keep healthcare providers from attending to their duties. This means that we could not ask for a lot of information that might have impacted HCW overwork during the pandemic. For example, we could not gather data on pre-pandemic attendance of HCWs and, therefore, cannot ascertain baseline levels of gender differences in work attendance. We also could not collect information on Personal Protective Equipment (PPE) and therefore could not assess if the ‘one size fits all’ approach towards designing PPEs contributed to gender differences in work attendance. We also did not collect information on the sub-specialties of healthcare providers, which might have influenced their work attendance. Finally, we also lacked variables on personal or household exposure to COVID-19, the number of children, school closures, dual provider households, and parenting requirements, which might further explain household burdens as a mediator in the relationship between gender and overwork.

In sum, these findings expand prior research before the pandemic and largely from more resourced settings by showing that males are more likely than females to report HCW overwork. This is partly explained by women's greater domestic responsibilities in this context. Given the heavy reliance on women HCWs to sustain frontline care generally and in situations of crisis like the pandemic, health systems cannot afford to leave this gender inequality unchecked. Hence, domestic labor support for HCWs may be a useful means of building more equitable hours of care for providers in this context. Longer-term social norm shifts in households and health systems can further support female HCWs to provide care, strengthen health systems, and advance paid employment and leadership opportunities for a more gender-equitable health care workforce. Most importantly, we must bolster our healthcare systems with larger numbers of HCWs across cadres and move away from sex-disaggregation of these cadres; this effort is likely the one most rapidly needed to rebuild our ravaged health systems globally.

## Contributors

Modupe Taiwo: Conceptualization, funding acquisition, validation, project administration, writing-original draft, writing-reviewing & editing

Oluwatoyin Oyekenu: Investigation, project administration, resources, supervision.

Ferdinard Ekeh: Investigation, project administration, resources, supervision, data curation, software.

Arnab K. Dey: Formal analysis, writing-original draft

Anita Raj: Conceptualization, funding acquisition, writing-original draft, writing-reviewing & editing.

The raw data was assessed by Ferdinard Ekeh and data analysis was undertaken by Arnab K. Dey

## Data sharing statement

The data that support the findings of this study after deidentification are available from the corresponding author upon reasonable request. Investigators whose study protocol that proposed use of the data has been approved by an independent review committee can access the data.

## Declaration of interests

All authors declare that they have no conflicts of interest.
